# Trance and Trancoidal States in the Lower Animals

**Published:** 1881-04

**Authors:** George M. Beard


### ART. VIII.—TRANCE AND TRANCOIDAL STATES IN THE LOWER ANIMALS.

TRANCE is a concentration of nervous activity in some one direction, with corresponding suspension of nervous activity in other directions.

This state can be induced in all animals, higher and lower, where there exists even the rudiments of a nervous system.

Trance is a purely psychological state, and is to be studied by psychological methods ; but it may be induced either psychologically or physiologically, or by both combined. We can only arrive at a true conception of its nature and its laws by psychological reasoning and observation ; and the study of the subject belongs preëminently and exclusively to psychologists. The physiologists of the world, either in this or preceding generations, have not, as a rule, experienced interest in the phenomena of trance, and when they have attacked its problems have failed, if not entirely surrendered to delusions. In this organized stage of trance, into which we are now entering, the subject must be developed mainly or exclusively by psychologists.

It has taken several centuries of slow evolution of ideas on this subject to bring it out of the stages where it was supposed to be the property of non-experts in all science ; next of scientific men in general, including physicists, astronomers and botanists ; next to surgeons, next to physicians, next to general physiologists, next to special physiologists, next to neurologists—students of the nervous system ; and finally, to that special department of neurology called psychology, by which it is to be organized, made verifiable and viable, and is to become the common and permanent possession of mankind. All sciences have passed through these stages, either slowly or rapidly ; the history of trance is not, in this respect, any way peculiar.

Trance in the lower animals, as in man, can be induced by various procedures, which in their various permutations and computations may be numbered almost by thousands, and the belief, almost universal among scientific men, even those who have given much time and labor to these phenomena, that there is some peculiar, definite, and circumscribed virtue in any one procedure for the inducing of trance in animals, is as unscientific, and as far out of harmony with the facts, as is the same principle in regard to inducing trance in man. And as in man, the form of trance to which attention has been longest and most frequently directed—induced trance—is, when broadly and philosophically analyzed, but a part and a fractional part of trance in general, and one of its varieties only, and to be scientifically studied only in its relation to the other varieties and as parts of the great whole, so in the lower animals, trance induced by any of the methods and processes hereafter to be noted, is but a variety of the general trance state to which lower animals and higher animals are alike liable; and the same law governs all the phenomena, whether occurring in man or in the animals beneath him, or whether brought about by any manœuvrings or manipulations, or occurring spontaneously in health or disease.

To study trance in the lower animals separately from trance in higher animals is indeed scientifically impossible; since, if we do not understand this trance state in any one animal higher or lower, we cannot understand it in other animals; but if we do understand it in any one animal, however low in the grade of the animal world, we can also understand it in its general principles in all those that are above it. The more complex the phenomena of life in any animal out of trance, the more complex the phenomena of life in that animal when in trance. Hence, trance in man is, out of all comparison, a more valuable scientific study than trance in any of the lower animals; and trance in finely organized, intellectual and sensitive natures is, in its manifestations, far more rich, imposing, and valuable than in the coarse, dull, and phlegmatic. The special subject, then, of trance in the lower animals is of interest chiefly as a part of the general subject of trance in the higher animals; just as insanity or epilepsy in the lower animals are of but little interest save as parts of the general scientific study of epilepsy and insanity; and to begin and to end our study of trance by experiments on animals alone, as some have done, is most unscientific, unwise, and unsatisfactory. If trance could be induced in animals alone, and not in man, we should never know anything of trance; it is only by reasoning downward from the phenomena of trance as they appear in man, that we can be able to understand and coördinate the same phenomena as they appear in the lower animals. Trance, indeed, like insanity, could not be understood at all if it were studied in animals alone.

Among the many influences or combinations of influences that are employed to disturb the mental equilibrium of an animal, so as to cause increase of nervous activity in some one direction and corresponding suspension of nervous activity in another direction, so as to put him in trance or the trancoidal state, the following are the most familiar, accessible and usuable.

1st. Acting on the emotion of fear, by reducing the animal to helplessness, by tying or confinement of some kind. This method is very effective, when united with some movement or manipulation that makes a strong physical impression, as when we turn a chicken's head under its wing, or place a horse on his side with one of its legs strapped, so that he is unable to help himself, or take hold of a crab in such a way that it cannot defend itself. The voice and the eye may reinforce the tying and confinement.

2d. Making strokes or passes on, over, or near an animal. The effect of these manœuvrings and manipulations gave rise to the delusion of animal magnetism, and by the same process of reasoning, gave rise to the same delusion in regard to man.

3d. Staring—fixing the eyes steadily on the eyes of an animal.

This is one of the methods by which lion-tamers succeed in preserving their power over wild beasts in their cages. In England, this last year, I saw somewhere the picture of a celebrated lion-tamer on the point of being torn to pieces in the cage of a leopard. The story is that this tamer never allowed himself to take his eye off this leopard, which was more wild than any other animal under his care; but the paw of the lion happened to fall on his shoulder as he was sitting down, and threw him off his balance, so that his eyes were for an instant taken off the wild beast before him, and this gave the animal opportunity to jump on him and destroy him before he could be rescued.

Why it is that the simple process of staring or gazing at an animal or a man produces trance or a trancoidal condition in the object stared at, is a psychological question of much importance in its bearing on this whole subject. This process is only one of very many processes, or variations of processes, that may be used to produce that disturbance of the mental equilibrium known as trance; but it is one that has been very much employed, especially in the entrancing of animals, and it enters more or less as a constituent into very many other processes.

The eyes of a corpse have no power, but the eyes of a living creature have great psychological power of a purely subjective sort on the living object on which they are fixed; hence the expression, "Evil eye," so familiar in the history of witchcraft.

The brightness of the eye, united with the variations in expression, corresponding to the variations in the mental emotions, are the elements which produce this effect of one living being over another living being; the fixed gaze by which the mind of one being, so to speak, shoots out through a bright, shining, glistening instrument, to the eyes of another being, exerting, on simple psychological principles, an influence of a most powerful character, and produces, or is liable to produce, either full trance or a trancoidal state; hence the expression, "If I can only catch his eye, I can succeed."

The study of the morbid fears of neurasthenia, or nervous exhaustion, is instructive on this point. Sufferers from the fear of man—anthropophobia—cannot look any person straight in the face; they can look at any other object—at any inanimate thing, it may be—but they cannot squarely face a human being of adult years for whom they feel respect, simply because of the loss of cerebral force. As a person thus afflicted becomes stronger, he finds that he can look directly into the eyes of any person.

Even a person in good health, if he is moderately sensitive, cannot look any one in the face for a long time, with ease, provided the other person is conscious that he is being looked at by him. In large audiences everybody looks at every other body, but no two persons look at each other long at a time. We select a person to look at who is not looking at us; and only in this way are we able to see each other; hence the habits of staring and glaring become the symbols of bad manners.

The above is the psychological elucidation of the long and well-known fact of the power of the eye to control animals, even the most savage and ferocious.

I am told by Dr. M. Josiah Roberts, that in hunting alligators, and sometimes, also, in hunting birds, in Florida, he found it possible to fix his eyes upon the animal or the bird, and to approach them so steadily while keeping his eyes thus fixed, that he could come very near them before shooting.

It is one of the conditions of all experiments of this kind, that there be no outside disturbing influences; the hunter must approach quietly, without making any noise, and there should be no noise from any other source. In experimenting with dogs, cats, and fowls, it is usually important, if not even necessary, to have the room still, and for the spectators to remain as quiet as possible while the experiment is going on; and even after the animal is entranced, the opening or closing of a door, or even a less noise, may cause the spell to be broken.

The reason for all this is clear enough: the concentration of nerve-force of the animal in some one direction which is brought about by gazing, or fixing the eyes upon it, is interfered with by the excitation of any other sense of a different kind from that which the operator is using. This would be expected from our theory of trance.

Even in experiments with human beings, where the subject is not perfectly trained, any noise on the part of the bystanders will spoil the experiment, and on the same principle.

4th. Bringing a bright light in sight of an animal.

This is a very effective procedure for the producing of trance, or a trancoidal state, in almost all kinds of living things that are endowed with nervous systems. This gazing is itself a concentration of force, which is the very essence of trance. Like all concentrated effort, it produces speedy exhaustion, which is often relieved by sleep.

The same method is very effective also in man.

5th. Music, either of the voice, or of instruments.

The power of music to charm animals has been well known for thousands of years, and, like the power of snakes to charm birds, has been observed and analyzed years before the scientific study of trance was thought of.

Thus we see that anything that causes concentration of nerve-force, through any of the emotions, especially of fear, through the general sensation, or through the nerves of special sense, trance or trancoidal states may be produced in animals just as in man. Man, however, can do what animals are not sufficiently intelligent to do, that is, keep from going into trance by voluntarily concentrating the mind in some other direction while the experimenter is operating on him. Any of my subjects, however well and long trained, can keep me from putting him into trance by simply thinking persistently of his mother or sister, or of any other person or thing that can be imagined. This I have proved by many experiments.

*Trancoidal States.*—Trance is a matter of degree; here, as everywhere else in nature, there are no leaps, jumps or surprises, but in a strict scientific sense, only evolution and development, each process preparing the way for another. There are, therefore, all shades and gradations of the trance state, from the complete trance, simulating death, down to a trifling and momentary fascination, and just as we have epileptiform, neurasthenoidal, or insanoidal states in man, which are in the neurasthenia, or insanity, so we have trancoidal states of all directions of epilepsy, gradations, from the highest to the lowest, scattered all along the road that leads to the full and complete trance.

TRAINING HORSES.

*Rarey's Method*—Charles Hall, M.D., and veterinary surgeon, gives me the following experience, illustration of Rarey's famous method of training horses: He was in the State of Missouri a number of years ago, stopping at the cabin of a friend, and he was challenged to ride a filly with a bad reputation, that no one of those western experts in horses could ride. The horse would throw every one who attempted to ride it. Dr. Hall went to the filly as she was led out by three or four men, and they held her while he applied a strap to the near fore-leg below the fetlock, and then flexed the leg and fastened it in that position to the upper part of the leg; then gently fastened the surcingle to the body; then very carefully he fastened another long strap to the off fore-leg below the fetlock, and passing this long strap between the surcingle and belly of the horse, he told the men holding the horse to get out of the way. Then tying the bridle reins short over the neck of the horse, and taking a short, firm hold of the longer strap with his right hand, he gently pushed the horse to the right off her balance, and she fell upon her knees. The horse struggled furiously for three or four minutes, then reared, landing on its knees after every effort. In about ten minutes the horse became nearly exhausted, and then he gently pushed it over on its side. Then he fastened the off fore-leg in the same manner as he had fastened the near fore-leg.

Then the horse, after resting a few moments, began again to rear, and struggled to get free, landing on his knees every time he reared as before. While the horse was resting, Dr. Hall stroked him gently, and after a while he put a saddle on him and got on him; the horse's legs being still fastened in the manner described, but all the while proceeding with the greatest gentleness. At length in a few moments he unfastened the near fore-leg, and induced the filly to get up on three legs, the doctor remaining on its back. The doctor then dismounted and mounted two or three times, the filly standing on three legs and not offering to stir from its position. Then the doctor again strapped the near fore-leg and laid the filly down as before; then talked soothingly, patted her, and put the saddle and bridle off and on two or three times; then, taking off both straps, he started on a ride of four miles without any difficulty. The neighbors looked on in astonishment, expecting that the horse would conquer, as he had conquered all others who had tried to ride it. The next day the filly threw him once, although on but three legs; he never attempted it afterwards. It became a good horse; so impressible did it become that on merely touching the fore-legs the horse would immediately lie down. If a rope be fastened in the mouth of a horse and around the lower jaw, and the other is fastened around his neck, the horse becomes helpless, and feels his helplessness, and will follow his master round and round like a child. This experiment has been done in my presence by horsemen.

*House's Method.*—Dr. House has kindly exhibited to me his method of operating on the mouths of horses. I have seen him go up to a horse that was famous for biting, and put his fingers, hand, and arm up to the elbow in his mouth, and manipulate at leisure; the horse apparently relishing it, certainly showing no sign of anger or dissatisfaction; and this he does with equal ease on a horse he has never seen before.

It matters not how wild, furious, ugly, or unmanageable the horse may be. It is not necessary that the horse should be tied or held; but it is more convenient to have him in a stall where he cannot run away.

Dr. House further tells me, that savage horses frequently rush at him, but that he always stops them by his voice and manner. He also castrates horses without using anæsthetics, or confining them. He tells me, also, that he has no doubt that he could whip a horse to death, in the open field, where he was not confined or tied in any way. The secret is to approach the horse suddenly, and with a vigorous manner, and with a loud voice, strike him a blow so powerful, that he is paralyzed with fear, and cannot move, just as he is liable to be paralyzed while standing on a track as a train is approaching. Dr. House professes, also, to be able to subdue horses as well as Rarey could, without the appliances that Rarey used.

Through all these performances on horses, or elephants, or dogs, or hens, or crabs, or geese, or human beings of a high or low organization, there is one common principle, which, when once fully understood, reduces all to consistency, unity and clearness, viz.: the feeling of helplessness and subjugation in the animal operated upon.

When once that feeling is produced by the eye, by the voice, by manipulations, or by the sudden infliction of pain, the operator becomes the master, and can do with the animal as he pleases.

In order to become the master, the operator must feel that he is such, and act accordingly, else he will fail. Practiced operators abstain instinctively from all manifestations of fear, or even doubt or anxiety.

The horse is a timid, and, save in narrow lines, as in memory of places, a stupid animal, else he could not be so easily frightened and subdued.

*Elephants.*—Mr. W. H. Cross has given a lifetime to the study of elephants in their native countries and in menageries, tells me that these animals are subdued in the same way that Rarey subdues horses, but, being bolder and stronger, and more intelligent, require more time and force for their subjugation. By ropes and pulleys their fore and hind legs are stretched out so that their bellies rest on the earth, and by another rope with pulleys are cast on their side. In this position they will remain for hours, sometimes for days, before they are subdued. The sign of submission is a yell or scream, and it is unsafe to unloosen them before this sign is given. Mr. Cross declares they are governed wholly by fear, and not at all by love; they have no affection for their keepers, and as a law it may be stated that all wild animals ever controlled by man, must be subjugated and kept in snbjugation through the emotion of fear.

In 1839, Dr. Wilson, of England, published a work on the effects of trance on the animals in the London Zoological Gardens. He speaks of experiments made on the following animals: horses, cows, calves, leopards, a lioness, cats, dogs, pigs, horses, fowls, turkeys, ducks, geese, maccaws, fish, roach, dace, gudgeons and loach. The method which he employed was to make passes, in the old style. The time required to produce trance sleep varied much in different animals, and in the same animal at different times, all the way from five minutes to an hour. With pigs, from three-quarters of an hour to an hour was required. There were many failures in all these experiments.

The effect on dogs were at first, restlessness, playfulness, stretching, yawning or trembling. On cats there was irritability, sometimes quarrelsomeness. He says that the lioness stopped eating, and grasped a joint between her jaws and kept it in her mouth without letting go her hold for twenty minutes ; her eyes were closed only at short intervals. On leopards the experiments were failures. One of the elephants was put to sleep in five minutes ; previous attempts, kept up for an hour, made him nervous and irritable.

Those who attempt these experiments, either on the savage animals of the menageries, or on the animals of the household, will meet with more failures than successes, just as with similar attempts on human beings. Indeed, the failures are as much a part of law as are the successes, and are not mysterious. It was quite clear, that in these experiments of Dr. Wilson, the animals were not thrown into a full trance as a rule, but into a trancoidal state. In some of the animals, as pigs, the excretions were affected, and there was sweating about the ears and neck.

The experiments of Czermak, which have been recently repeated to a considerable extent in Germany, England, and in this country, and the facts of which have for centuries been known to the laity, need not be referred to here, except to enforce attention to the fact that the phenomena are obedient to the same laws as similar experiments on any other animals or in man.

In experiments on dogs, cats, and guinea pigs, we must expect to meet with many failures ; and we may get out of patience before the animal shows any sign of geting into a trancoidal state. With all these animals, the methods of producing this condition are very numerous : making chalk lines, holding coins or other bright objects before them, pointing the finger closely at them, striking the back, or tickling behind the ears, strapping or confining their limbs, and then striking them, or pointing at them, etc., etc., etc.

With hens, the success is more uniform according to my observations, than in dogs or cats or pigs. The old experiments of placing the head of a hen under her wing, and then tossing it about for a moment, and afterwards laying it down on a table, is one that can be repeated by anyone, and with results that are pretty constant; similarly the experiments on crabs or guinea pigs give pretty uniform results.

*Jack Shooting of Deer.*—In this country it has long been the custom to shoot deer at night, by the aid of a strong light on the hat of the hunter or on the prow of the boat. I have several times had an opportunity to engage in this amusement in our northern woods.

The conditions are the light boat, with the boatmen in the stern who pulls in a noiseless manner; the hunter in the bow of the boat must also keep perfectly still, holding himself in position to be in readiness to shoot whenever the deer at the water's edge becomes dazed by the light. This dazing is a trancoidal condition, although not full trance; but it is quite sufficient to keep the deer from doing precisely what would be the best thing for him to do, and what he would do if he had full control of his faculties—run away—and compels him to remain still until he loses his life. Other animals are sometimes hunted in the same way.

Fishing with torches on the boat, and the tendency of moths and other insects to drive themselves furiously against the light, have a similar explanation.

The familiar habit of catching fish by tickling, is also an illustration of what we are here describing. In New England, it has long been the custom to tickle suckers until they become insensible, and then catch them; and trout are frequently caught in this way at certain seasons of the year.

The Iguana, a species of lizard in the Bahamas, is caught by the following process: A negro carries a long cord with a whipcord at the end; on discovering the game on the limb of a tree, he begins to whistle; the animal stretches out his head, seeming to listen intently; the negro continues to whistle softly, cautiously advances, and tickles the animal with the cord; the animal seems pleased with the process, turns on his back, stretches himself out like a cat before the fire, manifesting the most perfect contentment. Soon the negro, by a sudden jerk of the cord, brings him to the ground.

It is stated that turtles will sometimes come out of the water at the sound of whistling, and crawling on logs, remain there as long as the whistling continues; stretching out their heads as far as possible, showing every appearance of enjoyment; and it is also claimed that at such times one can handle them without difficulty.

*Trance in the lower orders of Man, as compared with Trance in the lower Animals.*—The philosophy that I have tried to make clear in all my writings on this subject during the last few years, is that trance is a generic state, applicable to all living beings endowed with nervous systems; that, in short, it is unity, whether existing in the lower orders of man or in animals, or however induced. He who does not start out from this point, can never reach the truth on this subject of trance. What the phenomena of trance are among intelligent men and women, I have elsewhere, and many times, described in detail.

Experiments on the *lower* order of human beings have not, so far as I am aware, been studied in a thorough scientific manner.

On the islands of the South, between Charleston and Savannah, there are thousands of negroes who, from their geographical isolation, have been at no time brought in contact with any considerable number of whites, and who, therefore, in their intellectual development, represent the primitive negro with some slight improvement.

I have had good opportunities for studying these people, and have found their psychology in many ways interesting. Their language, although professedly English, is so different from ours, that it is difficult for a stranger to converse with them. We do not understand them, and they do not understand us; and the vocabulary of the majority of them is limited to a very few hundred words. They seem to be almost as superstitious as their African ancestors, despite the influence of the Christian religion, and their opportunities for attending school since their emancipation. They have the capacity neither for forming general ideas, nor for carrying two ideas in the brain at the same time ; in all respects they are dull, slow, short-sighted and unambitious, quite inferior to the style of negro sometimes seen in the North, and in other portions of the South.

I had some curiosity to see how trance experiments would work with these people, who may be regarded as somewhere between the higest order of animals and the lowest order of men; and this year, while making a short visit to these islands, I subjected quite a number of them to experiments. In order to get a chance to make these experiments, I caused notice to be sent around among them, by one of my friends, that a "shout" would be held in the barn. "Shout" is the negro term for an African dance of a peculiar and monotonous character, united with singing. It is a very popular amusement with negroes of the South, and when the chance is offered with a cheap entertainment added, they will keep it up all night.

Quite a large number assembled at the appointed hour in the evening, and I subjected several of the young ones to the experiments, but met with considerable difficulty, from the fact that I found it impossible to make them understand what I wanted. Some of them professed to be asleep, in order to deceive me, but in a very bungling way. I placed a number of them on a bench, and directed them to look steadily at a bright light. They did so, and in about ten minutes one of them became so thoroughly entranced, that I had him taken off the bench and removed to another part of the room. The "shout," a very noisy performance, then went on, but he did not waken. This greatly astonished the people, who gathered around, and they manifested much superstition in regard to his condition. While in this trance-sleep the pulse was natural, the eyes, as usual in such cases, resisted the attempt to open them, and rolled upward; and the whole expression of the face was that of one in profound sleep ; but it differed from normal sleep in this, that it was more profound, and we had some difficulty in arousing him. On coming out of the condition, he looked about with a dazed expression, and asked how he came from that bench, showing that he had been unconscious from the time he went into the sleep, and knew nothing of being carried from the bench to the place where he was lying.

This primitive negro was entranced in the same way that animals are entranced, or human beings of any order of intelligence; by gazing upon the light, the nervous energy was concentrated in one direction, with corresponding suspension in other directions. I used that method as, on account of the slight degree of intelligence on the part of the audience, I could not, very well, use my numerical method—stating that, on my counting twenty-five, they would be asleep; they could not understand my language or pronunciation.

The effect of silence, as in the entrancing of animals, was noticeable in this experiment. While others were laughing and making a noise, there was no success; but as soon as silence was enforced, the subject went to sleep.

The facts on this whole subject of trance and trancoidal states in the lower animals, may thus be briefly summarized:

*First*—Trance is a concentration of the nervous activity in some one direction. It is a state of degrees and gradations, all the way from full trance in which there is absolute suspension of the nervous activity in every direction except one, and a corresponding concentration of activity in an exceedingly narrow and limited phase, as in apparent death and long-continued rigidity, and so-called trance coma, to the mildest and most transient dazing and bewilderment.

Trance, as it exists in lower animals, whether quadrupeds, fish or insects, or in forms of life in which the nervous system exists, is the same condition precisely as trance in human beings, and is explained by the same theory,

Through the whole range of phenomena, it is obedient to the same natural law, and which is now no longer mysterious. It is explained as satisfactorily as any of the great laws of nature are explained, certainly as any in the domain of biology.

The state of trance has indeed, in many of its aspects, already obtained the predictable stage—the last and best test of the organization of any science, where we can tell beforehand what will happen with almost, in many instances, unfailing certainty.

*Secondly*—The only difference between the milder trancoidal states so often observed in animals and in men, as in cases of intellectual absent-mindedness and the temporary loss of presence of mind, and the full trance in which the animal or person is absolutely unconscious for minutes, or hours, or days, or sometimes years, is a difference of degree rather than of kind; and there can be no scientific study of this subject which fails to recognize this fact.

These trancoidal states bear much the same relation to full trance that epileptiform and neurasthenic or neurasthenoidal states or insanoidal states bear to epilepsy or neurasthenia or insanity.

*Thirdly.*—The methods or processes of inducing trance and trancoidal states in the lower animals and in man are infinite, and there is no one of those methods that are best known that can be said to have any special or pre-eminent virtue over any other, except of convenience and degree.

The philosophy in all these processes and manœuvres is to so alter the nervous equilibrium as to produce concentration of the nervous forces in some one direction, with corresponding cessation of nervous activity in other directions, and this can be accomplished by acting on the nerves of general sensation, or by any of the special senses, or by several of these modes combined; and these purely physical processes may also be reinforced by psychological or mental processes; as when, for example, we act in any way upon the emotion of fear. Effects the most speedy and most powerful are obtained when we combine the excitation of the nerves of general sensation or of special sense with profound excitation of emotion of fear and induction of physical helplessness; as when a horse is cast on his side so that it is impossible for him to rise, or a hen is securely tied by the feet, and at the same time the emotions of fear are energetically acted upon, and the nerves of sensation are affected by manipulation and by gazing into the eyes. The simple excitation of the emotion of fear, is itself, without any physical accessories, the most powerful of any single exciting cause in animals or in men. When, for example, a horse is in a stable that has caught fire, he is unable to move; he is in a condition of trance, and the paralysis of motion which makes him burn to death rather than escape, is one of the symptoms of the trance thus induced.

The temporary paralysis of a horse unable to cross a track or move in any direction when a train of cars is approaching, illustrates the same principle. Human beings, when surprised in the same way, as when an alarm of fire takes place in a crowded building, are likewise entranced, and exhibit the same phenomena.

When theatres are burned, as in Brooklyn a few years ago, and very lately in Nice, with great loss of life, the audiences are largely entranced through the emotion of fear, reinforced by their helplessness; and those who die are unconscious before they meet a painless instead of a painful death.

Of the special senses, that of sight is decidedly the best to act upon in order to induce the trance or trancoidal state. All the other senses may be similarly utilized; the hearing, as when the animal listens to music, or to any monotonous sound, as the falling of water, or the frequent ticking of a clock or watch; the sense of smell, as when some powerful and agreeable odor is near to the nostrils.

All these experiments apply with equal force to the higher as well as to the lower animals.

*Fourth*—Trance induced by any of the numberless processes above described is but a part of and is obedient to the same law as all the other varieties of trance, including those which appear spontaneously, or through any physical or mental excitation whatsoever, as epileptic trance, alcoholic trance, somnambulistic trance, cataleptic trance, ecstatic trance. To study induced trance in animals, as distinguished from induced trance in man, or to study induced trance in man or animals apart from the general generic subject of trance, is unphilosophical, and has led, and is leading still, to confusion without limit among those who are investigating this subject at home and abroad.

The erroneous, and misleading, and self-contradictory terms that have been applied to this condition, such as "hypnotism,"

"Braidism," "somnambulism," "catalepsy," &c., &c., should, as rapidly as possible, be driven from the scientific literature of the subject, and relegated exclusively to the possession of non-experts and delusionists.

GEORGE M. BEARD, A.M., M.D.

